# Research Progress of Machine Learning in Extending and Regulating the Shelf Life of Fruits and Vegetables

**DOI:** 10.3390/foods13193025

**Published:** 2024-09-24

**Authors:** Dawei Li, Lin Bai, Rong Wang, Sun Ying

**Affiliations:** 1 College of Light Industry Science and Engineering, Beijing Technology and Business University, Beijing 100048, China; lidawei@btbu.edu.cn (D.L.); 2230402043@st.btbu.edu.cn (L.B.); 2 Alumni Association, Beijing Technology and Business University, Beijing 100048, China; 3 School of Computer and Artificial Intelligence, Beijing Technology and Business University, Beijing 100048, China; 2330602095@st.btbu.edu.cn; 4 China National Centre for Quality Supervision & Test of Plastic Products (Beijing), Beijing 100048, China

**Keywords:** machine learning, shelf life prediction, artificial intelligence, vegetables and fruits, food safety and quality, food processing

## Abstract

Fruits and vegetables are valued for their flavor and high nutritional content, but their perishability and seasonality present challenges for storage and marketing. To address these, it is essential to accurately monitor their quality and predict shelf life. Unlike traditional methods, machine learning efficiently handles large datasets, identifies complex patterns, and builds predictive models to estimate food shelf life. These models can be continuously refined with new data, improving accuracy and robustness over time. This article discusses key machine learning methods for predicting shelf life and quality control of fruits and vegetables, with a focus on storage conditions, physicochemical properties, and non-destructive testing. It emphasizes advances such as dataset expansion, model optimization, multi-model fusion, and integration of deep learning and non-destructive testing. These developments aim to reduce resource waste, provide theoretical basis and technical guidance for the formation of modern intelligent agricultural supply chains, promote sustainable green development of the food industry, and foster interdisciplinary integration in the field of artificial intelligence.

## 1. Introduction

With the improvement of economic level, people’s demand for vegetables, fruits, meat, seafood, and other products continues to increase, and the market prospects are very promising. But equally, food also faces the risk of corruption and spoilage, which leads to significant waste of food and economic losses. Therefore, the importance of food shelf life for consumers and operators is self-evident [1]. According to the Food and Agriculture Organization of the United Nations, globally, about one-third of all food produced for human consumption is lost or wasted, approximately 1.3 billion tons [2,3]. The main reason for food waste is the deterioration and spoilage of food itself during storage or processing. Among the wasted food, the proportion of fruit and vegetable waste accounts for about 42% because the fruit and vegetable has a short shelf life and is susceptible to the external environment during storage and transportation. According to data from the National Bureau of Statistics, China’s fruit and vegetable production in 2022 reached approximately 1.091 billion tons, of which fruit production was about 300 million tons and vegetable production was about 7.91 tons. The loss rate of fruits and vegetables in logistics links such as picking, transportation, and storage in China is around 25–30%, while the loss rate of fruits and vegetables in developed countries is controlled below 5% [4]. So reducing the spoilage and prolonging the shelf life of fruits and vegetables has become one of the current research hotspots. The shelf life of food refers to the longest period during which the quality and safety of food simultaneously meet the requirements for consumption and meet different evaluation indicators. The evaluation indicators mainly include nutritional indicators, hygiene indicators, and consumers’ sensory evaluation under the premise of food safety. Accurate prediction and calculation of the shelf life of products under specific conditions can not only provide a basis for the selection of product storage conditions and transportation modes but also provide a reference for exploring the possibility of further extending the shelf life of food by improving product formulation and processing technology [5]. The traditional methods for determining food quality and shelf life are mainly based on microbial detection methods, physical and chemical indicator detection methods, and sensory evaluation methods [6]. However, these methods require the determination of standard procedures, which is destructive to the product to a certain extent. Sensory evaluation requires a large amount of human involvement and also carries a certain degree of subjectivity, which undoubtedly increases the cost and complexity of the food quality measurement process and brings error to the measurement results [7,8]. The existing food shelf life prediction technology usually monitors and regulates the environmental parameters where the product is located in order to determine the shelf life of the food. There are two main methods for determining the shelf life of food: one is to describe changes in food quality based on dynamic equations or mathematical models involved in biochemical indicators during food storage and then determine the shelf life of food. Although this method can clarify the quality changes during food storage, the results may not be satisfactory under specific products and complex environmental conditions [9]. A machine learning approach is a data-driven technique that learns and predicts future outcomes from training data. Instead of predefining explicit rules or algorithmic processes, this approach makes predictions by analyzing patterns and associations in large amounts of data. This means that machine learning models can adaptively adjust their predictions based on new data inputs, which is difficult to match with traditional methods [9]. Table 1 compares the advantages and disadvantages of traditional dynamic models and machine learning models.

**Figure 1:**
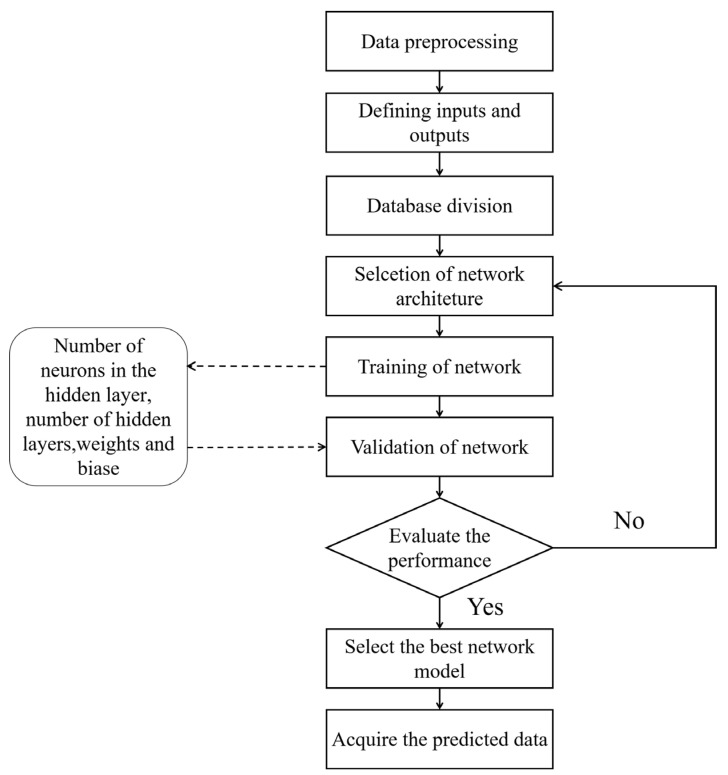
Process diagram of ANN model predicting shelf life of fruits and vegetables.

**Figure 2:**
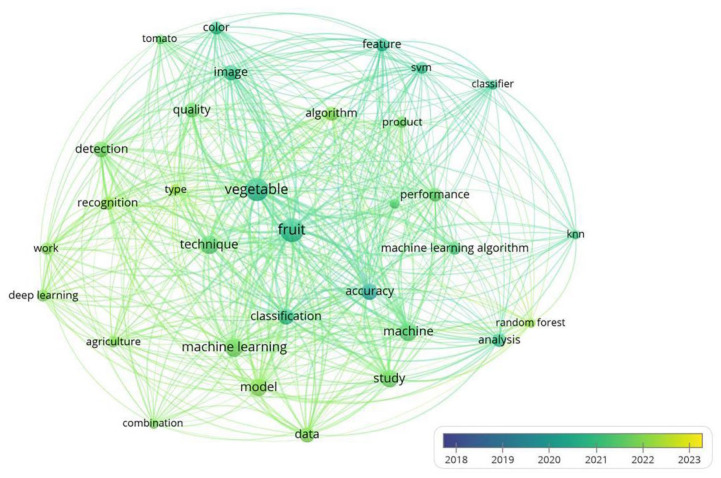
Overlay visualization of the title and abstract in references from 2018 to 2023. The result is conducted by VOSviewer version 1.6.19, Leiden University, Leiden, the Netherlands.

Machine learning (ML) refers to inputting available data or experience into a computer and, through programming, converting the input data into professional knowledge output or result output [14,15,16]. Its non-parametric nature enables them to simulate the nonlinear and dynamic processes involved in food quality changes during storage, accurately predicting the shelf life of food under diverse and fluctuating storage conditions by learning from experimental data [17]. Figure 1 shows the flowchart for using ANN models to predict the shelf life of fruits and vegetables [18]. It can integrate various types of data, including structured and unstructured data [19]. It can integrate various types of data, including structured and unstructured data. With its ability to analyze and process a large number of samples and identify complex patterns in high-dimensional variable spaces, it has been favored by researchers in the fields of food shelf life prediction and calculation. It is currently a widely used method in the field of shelf life prediction [20,21]. But at the same time, machine learning algorithms also have their own limitations, including the risk of overfitting, the need for large amounts of data, limited interpretability, vulnerability to adversarial attacks, and the challenge of hyperparameter tuning [22]. Using machine learning and food shelf life prediction as keywords, literature from 2018 to the present was retrieved from the Web of Science database. Based on the overlay of titles and abstracts of this literature, a visual data graph is shown in Figure 2. Figure 2 shows that the application of machine learning in fruit and vegetable preservation, including shelf life prediction and fruit and vegetable quality monitoring research, has recently received close attention from researchers (from 2018 to the present) and is becoming a research hotspot in the field of fruit and vegetable preservation.

This paper summarizes the widely used machine learning models in food shelf life prediction research, introduces the latest progress in the application of machine learning in food shelf life prediction and food safety, and describes the application of combining machine learning with other food quality detection technologies. This review aims to emphasize the important role of machine learning in food safety and encourage researchers to apply machine learning to monitor food safety and early warning in future research so as to promote the development of a high-quality food industry.

## 2. Machine Learning Algorithms Commonly Used in Food Preservation

**Figure 3:**
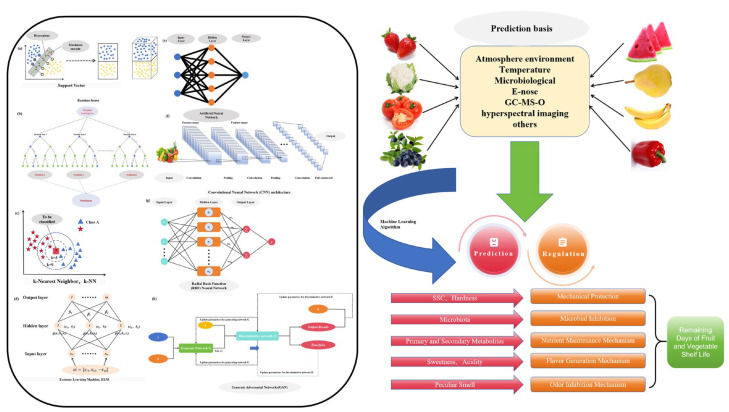
Schematic illustration of machine learning algorithms and their application in shelf life and quality control of fruits and vegetables: (**a**) support vector machine, (**b**) random forest, (**c**) k-nearest neighbors, (**d**) extreme learning machine, (**e**) artificial neural network, (**f**) convolutional neural network, (**g**) radial basis function neural network, (**h**) generate adversarial networks.

Due to its unique advantages of reliability, objectivity, accuracy, cost-effectiveness, and minimal time consumption, machine learning has been gradually applied in recent research to predict and judge food quality and shelf life [23]. Machine learning algorithms can be divided into traditional machine learning algorithms and deep learning algorithms. Traditional machine learning algorithms mainly include support vector machines (SVMs), decision trees (DTs), random forests (RFs), k-nearest neighbors (k-NNs), extreme learning machines (ELMs), and artificial neural networks (ANNs), etc. Figure 3 summarizes the structural diagrams of various machine learning algorithms and their applications in shelf life and quality control of fruits and vegetables.

### 2.1. Traditional Machine Learning Algorithms

Support vector machine (SVM), originally proposed by Cortes and Vapnik, is a traditional machine learning algorithm developed on the basis of statistical theory and mathematics. It has a wide range of applications, high stability, and is mostly used for processing classification and regression tasks [24]. SVM models can be used to classify the level of spoilage in fruits or vegetables by analyzing sensory or environmental data. First, sensory or environmental data are collected from fruits or vegetables, which may include environmental factors such as temperature, humidity, and light conditions, as well as the level of spoilage obtained through sensory assessment. The collected data need to be preprocessed, such as cleaning, denoising, and normalization, to facilitate the processing and analysis of the model. Next, meaningful features are extracted from the preprocessed data, which can be numerical, such as temperature and humidity, or subtypical, such as the degree of corruption. After that, the SVM model is trained using the extracted features. SVM maximizes the spacing between different classes by finding a hyperplane for classification. After the training is completed, the accuracy of the model needs to be evaluated, which can be performed by calculating the model’s accuracy, recall, and other metrics. Based on the evaluation results, the model can be optimized, such as by adjusting the parameters in the SVM (e.g., penalty coefficient C and kernel function) to improve the classification performance. Once the model training and optimization are complete, the SVM model can be applied to new fruit or vegetable samples to predict the level of spoilage based on their sensory or environmental data. The principle of SVM is to transform the low-dimensional input vector into the high-dimensional feature space. By finding an optimal hyperplane in the multi-dimensional space of the data, the distance of all data samples from the optimal hyperplane is minimized, that is, the error is minimized. The hyperplane can be represented by Equation (1):

(1) $$f(x)=\omega^{T}x+b$$

where $\omega^{T}$ is the weight vector transpose and b is the bias.

It aims to minimize structural errors and use linear, radial basis, polynomial, and sigmoid functions to solve nonlinear separable problems and make them as linear as possible. It exhibits excellent performance in performing recognition tasks and predictions in high-dimensional patterns [20,25]. Khoramifar et al. used a combination of electronic nose and machine learning modeling to predict the quality changes and shelf life of potatoes during storage. The results show that the accuracy of potato shelf life prediction by principal component analysis (PCA), SVM, and other methods is 78–96%, which is an economical, efficient, and rapid method to monitor the quality change of potatoes and predict their shelf life during storage [26]. This study provides a theoretical basis for better understanding the correlation between fruit and vegetable quality and external factors through machine learning and contributes to the development of new fruit and vegetable preservation technologies, as well as the prediction and extension of shelf life.

A decision tree (DT) is a sequential model that predicts response variables by creating a series of decision rules and presents the distribution of possible outcomes in the form of a tree. It is a tree structure composed of root nodes, internal nodes, and leaf nodes, which is generally suitable for classification and regression problems. Among them, the root node contains the complete set of samples to be classified, the internal nodes correspond to test attributes, the leaf nodes correspond to decision results, and the classification rules are represented by the path from root to leaf [15]. The algorithm starts from the root node and enters other sub nodes based on different sample values, and so on, until the sample attributes match the current node attributes or meet some other pre-set stop conditions [21]. Compared with other algorithms, DT has the advantage that it can handle continuous or discrete data, and the decision tree structure is simple and strong interpretability. In addition, DT itself is more suitable for processing large and complex data sets, the training sample size is smaller than other data mining and statistical methods, and there is no multi-collinearity influence, which can explore the mutual effect between variables.

Random forest (RF) is an ensemble learning method based on DT classifiers, which can be used to solve nonlinear classification and regression problems [27]. RF further introduces the selection of random attributes in the training process of DT based on decision tree learning, which aims to improve the prediction accuracy of DT and control overfitting. Breiman et al. cited random forests in point cloud classification problems in 2001 [28]. In the prediction process, the decision tree acts as the RF base learner. When making classification judgments on point clouds, weighted voting is used to count the classification results of all decision trees, and the final category of the point cloud classification is determined according to the number of votes counted. This avoids the impact of a single decision tree on the classification results and ensures that the trained classifier has good robustness and noise resistance [29,30]. RF has stronger generalization ability than SVM and can handle high-dimensional data without decreasing prediction accuracy. However, its training process is relatively time-consuming [31]. Wunderlich et al. used carbon dioxide (CO_2_) sensors, volatile organic compound (VOC) sensors, ethanol sensors, pH sensors, near-infrared sensors (NIR), and regression algorithms to develop a prediction model based on collected sensor data. RF and Extreme Gradient Boosting (XGBoost) were used to predict the shelf life of fresh pizza. By evaluating various model indicators such as *R*^2^, mean square error (MSE), root mean square error (RMSE), and mean absolute error (MAE), it was found that the accuracy of the RF algorithm was slightly better than that of the XGBoost algorithm, with an *R*^2^ value of 0.99, indicating that the goodness of fit of the model was close to perfect. Among the five sensors, the VOC sensor exhibits the best performance with the highest *R*^2^ value. This study provides a highly accurate and cost-effective method to determine the freshness of pizza, predict more accurately the date of spoilage and the best before date, and reduce food loss and waste [32].

K-nearest neighbor (k-NN) is one of the simplest machine learning algorithms. The basic idea of the k-NN algorithm is to search for k training samples that are most similar to the input samples in the training set database when the samples are input, and then select the dominant category among the k training samples for classification and prediction and output them [33,34]. The k-NN algorithm uses distance measurement to determine the similarity between historical data and the predicted eigenvector. The main limitations of the k-NN algorithm lie in its storage requirements and computational costs. Due to the need for the k-NN algorithm to store all training data points in order to calculate the distance to each point during prediction, its storage requirements increase linearly with the size of the dataset. In real-time food monitoring applications, if the dataset is very large, storage requirements may become a limiting factor. Meanwhile, the computational cost of the k-NN algorithm is also quite high. For each new test point, it is necessary to calculate the distance between it and all training points and then identify the k nearest points for prediction. This process is very time-consuming when the dataset is large and may affect the response speed of real-time food monitoring applications. However, these limitations can be addressed by the following scheme: first preprocessing large datasets: reducing the size of the dataset through techniques such as data cleansing, dimensionality reduction, or feature extraction to reduce storage and computation requirements. For example, dimensionality reduction methods such as principal component analysis (PCA) can be used to reduce the dimensionality of the data, or feature selection methods can be used to select the features that will have the greatest impact on the prediction results. Secondly, cloud-based storage is also used: storing datasets in the cloud extends storage capacity and improves data accessibility. In this way, even if the dataset is very large, it can be managed through cloud storage, and the storage resources can be dynamically scaled as needed. Hanif et al. used the k-NN algorithm and the electronic nose sensor dataset to predict the shelf life of rice. This experiment shows that the *R*^2^ of the k-NN regression algorithm is 0.7217, which proves that the regression model is similar to the actual value. The RMSE score is 3.8043, and a lower RMSE value means that there is a small difference between the actual and predicted values. This study provides new ideas and references for predicting the shelf life of rice and indicates that the combination of electronic nose systems and machine learning algorithms can effectively reduce grain loss and resource waste [35].

Extreme learning machine (ELM) is a simple and efficient single-hidden layer feedforward neural network learning algorithm proposed by Huang et al. Initially, it is used to deal with single-hidden layer feedforward neural networks, and later it is extended to Radial Basis Function (RBF) neural networks, feedback neural networks, generalized single-hidden layer feedforward neural networks, and multi-hidden layer feedforward neural networks. It has been widely used in disease diagnosis, traffic sign recognition, quality assessment, and other fields [36,37,38,39]. In ELM, the input weights and hidden layer bias of the network are generated randomly, which avoids a lot of iterative adjustment in the training process. Only the activation function and the number of hidden layer neurons need to be determined, while the output weights can be directly calculated by the least squares method. Therefore, the number of hidden layer neurons will directly affect the performance of the model [40]. ELM itself has the advantages of fast learning speed, strong generalization performance, small training error, and good general approximation ability under the premise of ensuring model accuracy [41]. Huang et al. proposed a new method for predicting fruit freshness based on multi-perception technology and machine learning algorithms for blueberry preservation. By monitoring environment gas information at different temperatures and combining it with different machine algorithms (BP, RBF, SVM, ELM). The accuracy of different algorithms is 90.87% (BP), 92.24% (RBF), 94.01% (SVM), and 91.31% (ELM). This study not only achieves the purpose of predicting blueberry freshness but also improves the automation, intelligence, and high precision of fruit freshness prediction, providing a more solid theoretical basis and data reference for the combination of fruit storage and machine algorithm [42].

In addition, there are many other machine learning algorithms, such as Partial Least Squares Regression (PLSR), Extreme Gradient Boosting (XGBoost), and Back Propagation (BP), that have been widely used by researchers for food quality monitoring and shelf life prediction [43,44,45,46].

### 2.2. Deep Learning (DL)

Artificial neural networks (ANNs) can simulate the functions of the human brain and neural system. It abstracts the human brain neural network from the perspective of information processing, establishes a simple model based on the nonlinear relationship between parameters and targets, and forms different networks according to different connection methods [47]. It consists of a large number of nodes (or neurons) connected to each other, and there are two types of connections between neurons: fully connected networks and partially connected networks, depending on whether the neurons in one layer are connected to all or part of the neurons in the next layer. Each node represents a specific output function, called an activation function. The connection between each two nodes represents a weighted value for the signal passing through the connection, which is called a weight. The output of the network varies depending on the connection method, weight value, and activation function of the network. And the network itself is usually an approximation to a certain algorithm or function in nature, or it may be an expression of a logical strategy. Artificial neural networks are usually divided into three layers, namely the input layer, hidden layer, and output layer. The input layer corresponds to the independent variable, the output layer generates the predicted values of the dependent variable in regression or classification problems, and the hidden layer is a node layer located between the input layer and the output layer [48]. When the input information is input, it is combined with the weight factor, and the neuron performs a weighted sum on it and passes the result to the transfer function to generate an output [34]. The advantages of the artificial neural network itself are as follows:

(1) It has self-learning functions. For example, when realizing image recognition, just input many different image templates and corresponding recognition results into the artificial neural network, and the network will learn to recognize similar images through self-learning function. The self-learning function is of great significance for prediction. In the future, artificial neural networks will provide economic prediction, market prediction, and benefit prediction for humans, which has strong reference value for current shelf life prediction research. Researchers can use appearance images or spectral images of fruits and vegetables as inputs to achieve perfect control of fruit and vegetable quality and accurate prediction of shelf life without damaging them. Ripeness plays a crucial role in the quality and shelf life of apricots. Mozaffari et al. collected laser backscatter images of apricots at 650 nm and established artificial neural networks (ANNs), partial least squares regression (PLSR), and principal component analysis artificial neural networks (PCA-ANNs) models. The extracted images were used as model inputs to predict the quality parameters of apricots. The results indicate that there is a high correlation between backscatter images and quality parameters during the ripening process of apricots. The laser backscatter imaging methods can successfully predict the quality characteristics during the ripening process of apricots, and the ANN model has more accurate prediction performance than PLSR [49].
(2) Equipped with an associative storage function. This association can be achieved using a feedback network of an artificial neural network.
(3) The ability to search for optimized solutions at high speed. Finding an optimal solution to a complex problem often requires a significant amount of computation. By utilizing a feedback-type artificial neural network designed for a specific problem and exerting the high-speed computing power of the computer, it is possible to quickly find the optimal solution [50,51].

Therefore, ANN has a universal and forward-looking use in ensuring the freshness and safety of products, improving the efficiency of quality control, and resolving potential disputes between buyers and sellers. However, overtraining and extrapolation of artificial neural networks can limit the predictive performance of artificial neural network models.

Machine learning as the main tool for data processing has been applied in various fields. Traditional machine learning techniques typically require manual feature extraction. With the development of hardware computing and storage capabilities, the ability of machine learning can be improved by adding more complex structures to achieve deep representation of data [52]. Deep learning as a branch of machine learning is developed based on traditional ANN models. It uses networks to extract features from large datasets and learns a deep nonlinear network structure to achieve complex function approximation and characterize input data [53,54]. Deep learning shows the powerful ability to learn the essential features of data sets from a small sample set. The essence of deep learning is to extract and learn more useful features by building machine learning models with many hidden layers and massive training data, so as to improve the final accuracy of classification or prediction. The similarity between deep learning and traditional neural networks lies in the fact that deep learning adopts a hierarchical structure similar to that of neural networks. The system consists of a multi-layer network consisting of the input layer, a hidden layer (multi-layer), and an output layer. Only the nodes of adjacent layers are connected, while the nodes of the same layer and cross-layer are not connected. Each layer can be viewed as a logistic regression model. The differences between the two are as follows: (1) Deep learning emphasizes the depth of the model structure, usually consisting of 5, 6, or even 10 layers of hidden nodes. However, traditional neural networks are mostly shallow structured algorithms, which have limited representation ability for complex functions under limited samples and computing units. When faced with complex problems, their generalization ability is limited to some extent [25]. (2) Deep learning clearly highlights the importance of feature learning, and its feature learning ability is stronger than that of traditional neural networks. By layer-by-layer feature transformation, the feature representation of samples in the original space is transformed into a new feature space, making classification or prediction easier [55,56]. Pedro et al. used deep learning methods to monitor avocados stored in three different storage environments and classified their ripening stages based on their common features. Using convolutional neural network models for training to identify different maturity indicators in order to predict the ripening stage and shelf life of avocados. The results showed that the final prediction accuracy of this method for maturity assessment was 88.8%, and the predicted shelf life was within 0.92 days of the actual shelf life [57].

Convolutional neural networks (CNNs) are a type of feedforward neural networks (FNNs) that involve convolutional computation, have a deep structure, and are one of the representative algorithms of deep learning [58]. The composition of CNN includes the input layer, convolutional layer, activation function, pooling layer, and fully connected layer, mainly used for recognition, analysis, or classification of images [25,59,60,61]. The convolutional layer is constructed by convolutional kernels, and its parameters need to be set and optimized based on actual samples and scenarios. The pooling layer is generally located between two convolutional layers [62]. The convolution layer learns the input features and is transmitted to the next layer after activation function. The pooling layer resamples the computational output of the convolutional layer, but the output features after convolutional calculation may have too large dimensions. Therefore, it is necessary to pool the output features to achieve data dimensionality reduction. The fully connected layer is used as a classifier or to generate numerical output at the end [56,62]. Based on the traditional sensory evaluation method, Liu et al. used 10 physicochemical flavor indicators (e.g., catalase, flavonoids, soluble solids) of blueberries as input data and sensory evaluation scores as output data. Three different prediction models were applied and compared: SVM, convolutional neural network (CNN), and long short-term memory network model (LSTM). The results showed that the accuracy of the CNN model was significantly better than the other models, with root mean square error and mean absolute error of 0.96 and 0.78, respectively [63]. Aherwadi et al. used bananas as experimental samples and created two datasets, which included three types of images: 700 images of mature, immature, and overly mature each. By using CNN as the dataset to construct the model, the experimental results showed that the accuracy of the original dataset in the CNN model was 98.25%, while the accuracy of the enhanced dataset was 99.36%. These studies provide data references for finding the best deep learning algorithm that can be used to predict fruit maturity and fruit shelf life quality and contribute ideas for subsequent scholars to the progress of relevant research on food shelf life prediction, which is conducive to the development of sustainable intelligent agriculture [64].

Radial basis function neural network (RBFNN) also belongs to feedforward neural networks [17,65]. RBF neural networks are used to solve function approximation problems and have the best global approximation ability. The convergence speed is fast, and the problem of local minima is overcome [66]. The radial basis function neural network consists of an input layer, a hidden layer, and an output layer. There is no connection between neurons in the same layer, and adjacent two layers of neurons are fully interconnected. The input layer neurons have no computing function, and the connection weight between the input layer and the hidden layer is 1. The input layer transfers the input value to the hidden layer, and the hidden layer neurons respond to the input through radial basis functions, mapping low-dimensional nonlinear separable inputs to high-dimensional linear separable spaces. RBF is good at handling classification and regression task analysis [67]. Compared with the new dataset, this network also has acceptable generalization ability; as long as there are enough neurons, it can estimate each complex function with the required accuracy [68].

The hidden layer of the RBF model is the radial basis function activation function, and the transfer function in the hidden layer is generally a Gaussian function. The formula is as follows:

(2) $$\varphi_{i}(x)=\exp(-\frac{||x-c_{i}{||}^{2}}{2\sigma_{i}^{2}})$$

RBFNN needs to select M hidden layer basis functions, and the smaller the distance between the input vector and the center point, the larger the output of the network. The size of the central point matrix is the number of neurons in the hidden layer s* the number of neurons in the input layer *r*. Each *i* corresponds to *i* so that different input information can be reflected by different hidden layer neurons to the greatest extent.

The output layer neurons perform a linear weighted combination on the output of the hidden layer. The final output is

(3) $$y_{j}=\sum_{i=1}^{M}\omega_{ij}\phi ({\left\|x-\mu_{i}\right\|}^{2}),j=1,2,\cdots ,p$$

Implement mapping from input data to output results.

At present, the RBFNN model has been applied to fish freshness prediction. Jia et al. established and compared the RBFNN and Arrhenius models to predict and evaluate freshness changes of salmon slices at different temperatures during storage. Through the determination of thiobarbiturate (TBA), total volatile basic nitrogen (TVB-N), potassium value, and sensory evaluation (SA) on salmon fillets, the results show that these models are suitable for predicting the freshness of salmon fillets, and the relative error of the RBFNN model is less than 6%, indicating the best predictive performance [69].

Generative adversarial networks (GANs) are an unsupervised learning algorithm that can be used to generate highly realistic data such as images, audio, and video. It has a wide range of applications in image generation, semantic segmentation, data enhancement, and other fields [70,71,72]. GAN consists of two neural networks, a generator and a discriminator. The generator randomly samples from potential space as input, and its output results need to mimic the real samples in the training set as much as possible. The input of the discriminator is either the real sample or the output of the generator. Its purpose is to distinguish the output of the generator from the real sample as much as possible. The generator and discriminator confront each other and constantly learn, ultimately enabling the discriminator to accurately determine whether the output result of the generator is true. A high-performance GAN application requires good training methods; otherwise, the output may not be ideal due to the freedom of the neural network model.

The above algorithms have shown strong potential in judging food quality and predicting food shelf life. However, most of these algorithms are jokingly referred to as “black boxes” by researchers because they are difficult to explain the relationships between various parameters, so their interpretability is relatively limited. In the future, we should increase research on the interpretability of machine learning algorithms and accelerate their learning speed or calculation accuracy.

## 3. Application of Machine Learning to Food Shelf Life Prediction

During the storage process of fruits and vegetables, various storage conditions (temperature, humidity), food characteristics, packaging, and seasons can all lead to changes in the shelf life of fruits and vegetables. The fluctuations of these factors in the food supply chain may cause microbial changes that could lead to unexpected food spoilage, waste, and economic losses, as well as food safety and consumer trust issues. In recent years, researchers have increasingly attached importance to the application and development of machine learning in food shelf life prediction. By collecting and analyzing various data (e.g., temperature, humidity, microbial content, etc.) of food products during storage, machine learning algorithms are used to build predictive models in order to accurately predict the shelf life of food products under different conditions. This helps food manufacturers to optimize the production process and develop reasonable storage and transportation conditions, thus extending the shelf life of food and reducing waste. Moreover, through machine learning technology, the shelf life of food can be managed more scientifically and efficiently, reducing the economic loss and waste of social resources caused by food expiration. At the same time, it also helps to improve the overall quality control level of the food industry and promote the sustainable development of the industry. Feng et al. [73] used the CNN-SVM algorithm to construct a dynamic prediction model for key indicators and a live salmon state diagnosis model. They predicted the quality indicators such as texture, color, and pH value changes of salmon after 10 h of waterless cold chain transportation, which is of great significance for improving the survival rate of salmon during transportation and providing effective and reliable survival assessment and transportation management for salmon [73]. Table 2 summarizes the application progress of machine learning algorithms in food shelf life prediction.

**Figure 4:**
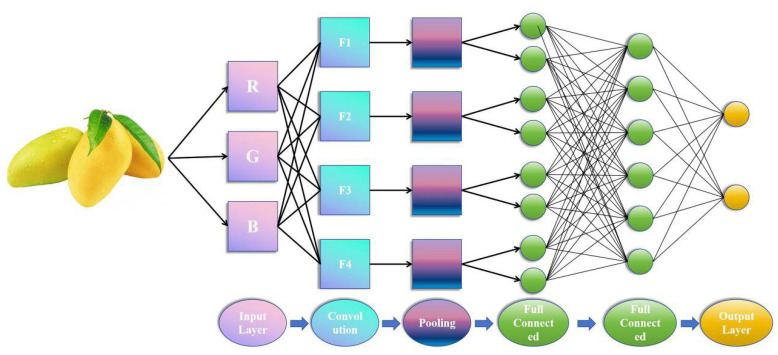
Prediction of Mango Shelf Life Based on RGB Imaging.

The process of shelf life prediction can determine the main evaluation indicators according to the characteristics of food, select appropriate models according to the type of indicators, and combine the experimental results to determine the shelf life of food. For each indicator, we can categorize them according to the source from which they were obtained. For example, indicators obtained by non-destructive methods include spectral indicators (visible spectrum, near-infrared spectrum) used to analyze chemical composition and ripeness, graphic indicators (color, shape, size, surface texture) used to assess the quality of appearance and ripeness, and environmental indicators (temperature, humidity, gas concentration) used to monitor the effect of storage conditions on shelf life. Whereas indicators obtained by destructive methods include chemical indicators (sugar content, acidity, pH, content of antioxidant substances), microbiological indicators (total number of colonies), and physical indicators (texture, color change, weight loss). Commonly used models mainly include models based on temperature change, microbial growth, and sensory evaluation indicators. The fast, accurate, efficient, and objective characteristics of machine learning can be used to effectively evaluate food, solve food safety problems, and reduce resource waste and economic losses. In the application process, by collecting a large amount of information and adjusting and training the model, parameters such as food storage environment, maturity stage, packaging type, freshness indicator, microbial indicator, etc. are used as inputs to the model, and the estimated shelf life is output, thereby reducing unnecessary resource waste and ensuring food safety [18]. As shown in Figure 4, researchers conducted RGB imaging based on visual inspection and mango external features, took them as input parameters of the model, and used the CNN model to predict the shelf life of mango [93].

### 3.1. Predicting Shelf Life Based on Storage Environment

The storage environment, such as temperature and humidity, atmosphere composition, light, and other factors, are closely related to the quality of fruits and vegetables, so they can be used as input parameters in the shelf life prediction process to accurately predict the shelf life of food. Generally, on the basis of a large number of research data, various environmental parameters and related limits suitable for the preservation of the food are determined, and then modeling, training, and optimization are carried out to quickly and accurately predict the shelf life of the food. Yu et al. [94] investigated the effects of maturity, storage temperature, and damage degree on the shelf life of Korla pears and established a prediction model using modeling methods such as backpropagation neural networks and generalized regression neural networks (GRNNs), achieving the optimal prediction of the shelf life of damaged Korla pears.

The results showed that maturity, storage temperature, and damage degree had a significant impact on the shelf life of pears and were inversely proportional to the length of shelf life. Damaged fragrant pears still have storage value and can be stored at low temperatures according to different degrees of damage to extend the shelf life of damaged fragrant pears. The results of this study provide theoretical guidance and data support for the selection of optimal storage conditions and the effective prediction of shelf life of fruits and vegetables, and also provide ideas for the prediction of shelf life of other fruits [94]. Zhang et al. studied the sensory qualities of apples during storage at 4 °C and 20 °C, including changes in flavor, texture, color, and taste. A shelf life prediction model was constructed by using partial least squares regression (PLSR) and artificial neural network (ANN) techniques. The results showed that low-temperature storage can effectively maintain the color, flesh hardness, and volatile compound release of apples. The acidity of apples stored at 20 °C decreases much faster than at 4 °C. Both models have achieved effective prediction of apple shelf life, laying a solid foundation for the development of fruit and vegetable preservation. The above research indicates that temperature, as one of the important factors affecting the shelf life of fruits and vegetables, has broad prospects as a model input in predicting the shelf life of fruits and vegetables. It is helpful for the development of new technologies for fruit and vegetable preservation in the food industry and effectively avoiding resource waste and economic losses caused by food spoilage [95].

Similarly, the gas components and proportions in the storage environment can also affect the preservation effect and shelf life of fruits and vegetables. Mohammed et al. used machine learning technology and multi-spectral sensors to predict the quality parameters and shelf life of fresh dates under natural atmosphere (control), vacuum-sealed bags, and modified atmosphere packaging (MAP), where the interior of the air-conditioned packaging has two different gas combinations: 20% CO_2_ + N_2_ and 20% CO_2_ + 10% O_2_ + N_2_. The author measured the shelf life and quality parameters (pH, total soluble solids (TSSs), sugar content (SC), moisture content (MC), and tannin content (TC)) of fresh jujube under different storage temperatures and times. Multi-spectral sensors were used to correlate fruit quality parameters with spectral analysis under the same storage conditions, and data sets were prepared to train prediction models. The results showed that vacuum sealing and 20% CO_2_ + N_2_ modified atmosphere packaging effectively extended the shelf life. And the prediction model effectively predicted the shelf life of fresh jujube, with *R*^2^ equal to 0.951. There is also a good predictive effect on other biochemical indicators of fresh jujube: *R*^2^ of pH is 0.854, *R*^2^ of TSS is 0.893, *R*^2^ of SC is 0.881, *R*^2^ of MC is 0.941, and *R*^2^ of TC is 0.909. The research results also demonstrate that gas composition and proportion can effectively affect food shelf life, and prediction models can predict food shelf life according to the atmosphere, which provides technical reference and literature support for the development and development of new prediction models [96]. Alden et al. studied the effects of modified atmosphere packaging (92% N_2_, 5% CO_2_, and 3% O_2_) and regular air packaging on the shelf life and post-harvest quality of cauliflower. The samples were stored at a temperature of 4 ± 1 °C and a relative humidity of 90 ± 5%. The results indicate that the shelf life of cauliflower packaged with modified atmosphere packaging can be increased to over 30 days. At the same time, the author also designed and evaluated multiple multi-layer perceptrons to predict the longest shelf life of cauliflower in modified atmosphere packaging based on the maximum acceptable color change. The mean square error (MSE) and determination coefficient (*R_2_*) of the artificial neural network (ANN) established were 0.0095 and 0.990, respectively, and the results showed that the shelf life of cauliflower under modified atmosphere packaging was up to 50 days [97]. These studies indicate that storage environment is a powerful factor affecting food shelf life. Researchers can establish models to predict the shelf life of food according to parameters such as temperature, humidity, gas composition, and proportion in the storage environment. In turn, the prediction results can be used to regulate the storage environment parameters so as to achieve the purpose of food preservation, extending food shelf life, and reducing resource waste.

### 3.2. Predicting Shelf Life Based on Physiological and Biochemical Indicators

The physical and chemical parameters of food are also the embodiment of food quality characteristics to a certain extent, so the shelf life prediction can also be carried out according to the physiological and biochemical parameters of food. Selvan et al. analyzed the effects of storage time and temperature (5, 25, and 45 °C) on the rancidity indicator and oxidation kinetics of grain particles and used an Arrhenius type equation to predict the shelf life of grain based on rancidity parameter data. The artificial neural network model based on a backpropagation algorithm was used to predict FFA, AV, and PV of grain particles. The results indicate that storage time and temperature have significant effects on free fatty acids (FFA), acid value (AV), and peroxide value (PV). Higher storage temperatures have a shorter shelf life. The grain has a shelf life of 8.5 weeks stored at 45 °C, while the grain has a shelf life of 7.5 weeks at 15 °C. And the model has a low root mean square error value (0.262) and a high *R*^2^ value (0.998), indicating high accuracy and persuasive results. This study showed that storage temperature and time affected the rancidity indexes such as FFA, PV, and AV of grains, and these rancidity indexes could be modeled using the Arrhenius equation and ANN model to predict the shelf life of grains [98]. Li et al. developed a shelf life prediction model for postharvest fresh grapes using radial basis function neural networks. Firstly, the final indicators that affect shelf life (storage temperature, relative humidity, sensory average score, peel hardness, soluble solid content, weight loss rate, decay rate, and color difference) are determined through correlation and significance analysis. Then, the weights of each indicator are calculated by using the Analytic Hierarchy Process (AHP), and shelf life times under different storage conditions are determined. The RBF network is optimized with training data. The results show that compared with the dynamic Arrhenius model, the backpropagation (BP) network, and the RBF network, the RBF network has the highest prediction accuracy. The maximum absolute error is 1.877, the maximum relative error (RE) is 0.184, and the *R*^2^ is 0.911. In addition, the *R*^2^ of this method for shelf life prediction of Italian grape and Red Globe grape were 0.853 and 0.886, respectively, and the fit degree was the highest among all methods, which also proved that the optimized RBF network was suitable for accurate shelf life prediction of different table grape varieties. This study not only provides a new method for predicting the shelf life of different grape varieties but also provides a reference for the quality and safety management of grapes during storage. It also provides experimental ideas and data support for the widespread application of RBF neural networks in predicting the shelf life of other fresh foods [76].

Machine learning has shown great potential in quickly and accurately predicting food shelf life. However, no algorithm can solve the shelf life prediction task for all foods. Each algorithm has its advantages and disadvantages, and different food types and input parameter choices can lead to different prediction accuracy. For example, CNN excels in the field of image recognition and is suitable for processing image data in food quality inspection. By automatically extracting image features, CNN can accurately identify the appearance defects and color changes of food products, thus improving the accuracy of food quality prediction. Its local connectivity and weight sharing properties make CNN efficient and robust in processing large-scale image data. However, SVM is good at dealing with classification and regression problems and is especially suitable for small and medium-sized complex datasets. It is advantageous for multi-category classification problems (e.g., different grades of food quality) in food quality prediction by finding the optimal hyperplane to separate different categories of samples. Therefore, it is necessary for researchers to compare the accuracy of different algorithms and choose the model with the best prediction accuracy and fastest learning speed as the ideal model to predict food shelf life. Using multiple machine learning methods at the same time may produce more accurate results than using only a single model to predict food shelf life [99].

## 4. The Application of ML in Extending the Shelf Life of Fruits and Vegetables

Machine learning is widely used for food shelf life prediction and extension due to its powerful computing power, high efficiency, and low cost. In recent years, researchers have also utilized machine learning technology to screen and determine key indicators that affect food flavor and quality and use the results as input for machine learning models to regulate food quality, so as to achieve the goal of extending food shelf life. In addition, the data obtained through infrared spectroscopy, electronic nose, electronic tongue, and gas chromatography and mass spectroscopy (GC-MS) can also be used as input or output for machine learning methods to predict food freshness. In machine learning models, data quality and variability have a crucial place, as the success of the model depends largely on the accuracy and diversity of the input data. Accuracy of data is the basis of machine learning model performance. If there are errors, biases, or inconsistencies in the input data, then the model will not be able to learn the correct features, leading to inaccurate predictions. Data diversity refers to the number of different features and variables contained in a dataset. A dataset with diversity can provide more information to the model, enabling it to learn more complex patterns and relationships and predict more accurate results. Therefore, this section reviews the application progress of machine learning in predicting food freshness or predicting food quality status through key indicators that affect food quality. Table 3 summarizes the application of machine learning models in food quality control or food shelf life extension.

These studies will help screen key indicators that affect food quality and shelf life, serving as inputs for machine learning models to improve model prediction accuracy in future research.

### 4.1. Screening of Potential Key Freshness Indicators

In the field of fruit and vegetable preservation, fruit and vegetable freshness is an important indicator of fruit and vegetable quality and is one of the most important decisive factors of consumer acceptance and preference, which has always been a focus of researchers’ attention. Therefore, accurately identifying the freshness of fruits and vegetables and ensuring their quality is an important part of extending and predicting their shelf life. The control of microbial communities, flavor, and quality during the storage process of fruits and vegetables is often used as an effective strategy to extend the shelf life of fruits and vegetables. But if manual control is required, these methods are usually time-consuming, labor-intensive, and costly. In recent years, machine learning has received widespread attention due to its powerful computing power, high efficiency, and low cost. By using machine learning combined with metabolome and transcriptome analysis, the correlation between differential genes and differential metabolites was obtained, the key microbial communities that affect the freshness of fruits and vegetables was clarified, and the key compounds for achieving the best flavor and quality of fruits and vegetables were identified, so as to realize the indirect regulation of fruit and vegetable quality. For example, You et al. used three spoilage indicators (pH, total viable bacterial count (TVC), and total volatile basic nitrogen (TVB-N)) as freshness indicators for lamb during storage and correlated them with the metabolite profile of chilled mutton to evaluate spoilage biomarkers in refrigerated brown mutton. The prediction model shows that 13 metabolites can be used as spoilage indicators for chilled mutton. D-glyceric acid, phenylalanine, methionine, glucose-1-phosphate, D-(glycerol-phosphate), lysine, ribitol, asparagine, and isomaltose were significantly (*p* < 0.01) correlated to the freshness indicators. A correlation coefficient with gluconic acid, citric acid, and trans-4-hydroxy-L-proline and the freshness indicators ranged from 0.539 to 0.911. 1, 5-anhydroglucitol was significantly correlated with pH (*p* < 0.05) and TVC (*p* < 0.01) [105]. In another study, researchers used weighted network analysis and random forest regression to identify biomarkers associated with changes in pork quality. The results indicate that random forests are an effective method for identifying biomarkers and predicting quality based on metabolites, and weighted network analysis provides the best parameters for studying metabolite interactions and elucidating meat quality changes [106]. These studies also provide new ideas and strategies for fruit and vegetable preservation. Researchers can use metabolomics to predict fruit and vegetable quality information and determine the shelf life of fruits and vegetables. Sara et al. recorded the metabolomics changes of strawberries under modified atmosphere and low temperature conditions during short-term storage and then predicted strawberries stored in different atmospheres based on partial least squares discriminant analysis (PLS-DA) statistical analysis to identify bioactive metabolites and determine the optimal atmosphere. The results showed that storage at 20% CO_2_+ 5% O_2_ at 0 °C for 48 h resulted in changes in primary and secondary metabolites, including sugars, organic acids (malic acid, oxalic acid, acetic acid, phosphoric acid), and phenolic compounds (flavonols, anthocyanins, phenolic acids). These compounds contribute to the enhancement of strawberry flavor, have great potential for improving strawberry quality, and are beneficial for human health [107]. Colonnio et al. used targeted metabolomic information from tomatoes and blueberries, as well as their sensory feature ratings, to establish statistical and machine learning models, predict the quality of both, and evaluate the accuracy of 18 different models. The results showed that the prediction accuracy of most parameters of tomatoes and blueberries was high, with a prediction accuracy range of 0.62–0.87. Among them, XGBoost, gradient enhancer, and RF have higher prediction accuracy than other models, and XGBoost has the highest prediction accuracy. In addition, the gradient enhancer model was used to infer the flavor compounds that contributed the most to each flavor attribute. This study predicted the flavor properties and quality information of the two kinds of fruits and vegetables, which contributed to the emergence of fruit and vegetable varieties with better flavor and better quality and indirectly realized the control of fruit and vegetable quality [108].

Therefore, according to the different types of fruits and vegetables, the primary and secondary metabolites and volatile flavor components of fruits and vegetables can be used as potential key indicators to characterize their freshness. This also provides new ideas and strategies for the research focus of subsequent researchers. At the same time, the clarity of potential key indicators affecting food quality is conducive to the improvement of fruit and vegetable quality and the extension of shelf life, which will directly affect the accuracy of the machine learning model, greatly improve the efficiency of the model, and greatly help consumers to make accurate purchase decisions and operators to make accurate supply chain optimization management strategies. Truly make contributions to national resource conservation and economic development under the current dual-carbon background.

### 4.2. Non-Destructive and Rapid Detection of Freshness of Fruits and Vegetables

**Figure 5:**
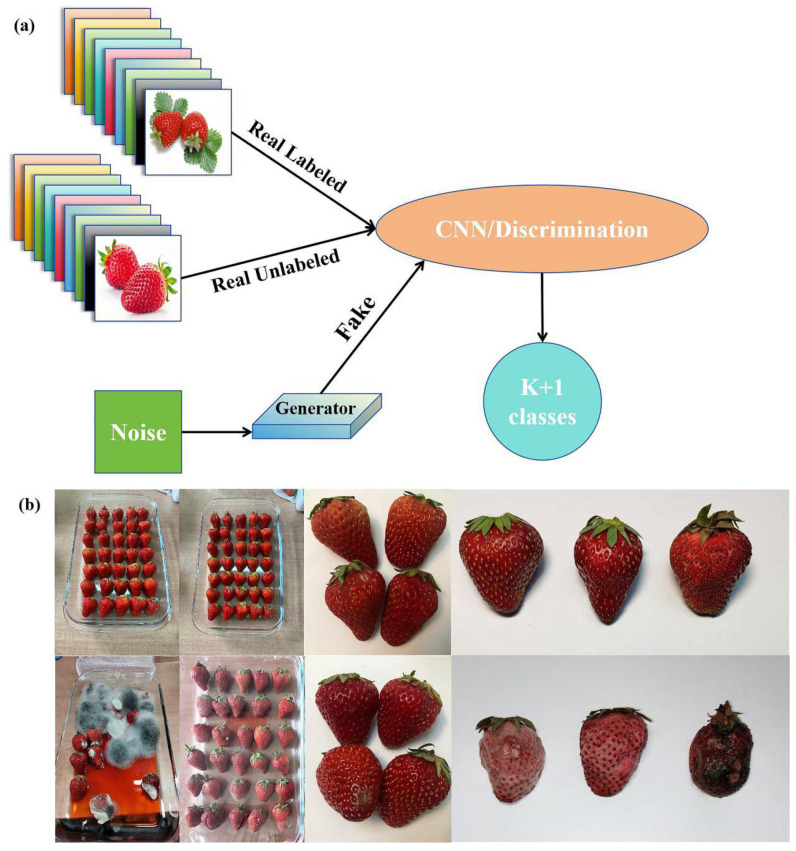
A semi-supervised GAN architecture for classifying strawberry freshness based on images and schematic diagrams of fresh and decayed strawberries: (**a**) A semi-supervised generative adversarial network (GAN) architecture for strawberry freshness classification. (**b**) The examples of fresh and rotten images.

At present, researchers also combine the non-destructive testing technology of fruit and vegetable freshness with the machine learning model, taking the results of non-destructive testing as the input of the machine learning model, and output the quality status information in fruits and vegetables after training the model with a large number of sample data. According to the difference in fruit and vegetable quality state information, the storage conditions and environmental factors of fruits and vegetables are regulated in turn to achieve the purpose of extending the shelf life of fruits and vegetables. The quality of fruits and vegetables is not only the focus of consumers but also the main concern of manufacturers and distributors. Classifying these fruits according to the ripening stage helps to adopt different methods for the preservation of fruits and vegetables at different stages. Benmouna et al. used hyperspectral information in the visible and near-infrared regions to perform non-destructive classification of the mature state of Fuji apples. First, the author collected the spectral information of 1000 apple samples at different ripening stages in the range of 172–450 nm, used a convolutional neural network (CNN) model to classify the samples, and compared the CNN model with three models based on artificial neural networks (ANNs), support vector machines (SVMs), and k-nearest neighbor (k-NN). The results showed that the CNN model is significantly superior to other methods, with a classification accuracy rate (CCR) of 96.5%, while the average classification accuracy rates of ANN, SVM, and k-NN were 89.5%, 95.93%, and 91.68%, respectively. This research is conducive to the development of new technologies for classifying fruits and vegetables at different mature stages and the formulation of appropriate preservation strategies for different fruits and vegetables, which could effectively ensure the quality of fruits and vegetables and avoid resource waste [109]. Figure 5 shows a semi-supervised GAN architecture for classifying strawberry freshness based on strawberry images and a diagram of fresh versus rotten strawberries.

In the same way, researchers are deepening their research on food quality monitoring. The research conducted by Lam et al. aims to provide a new solution for food monitoring. They propose an online non-destructive food monitoring method with self-powered capability, which utilizes deep neural network models to monitor changes in food quality and uses humidity sensors and gas sensors to monitor the total volatile organic compound content released during food spoilage in food packaging. Finally, the different states of food quality were predicted according to the monitoring results. In addition, the authors also utilized the sensor to monitor the quality of pork and fish in real-time for eight days under room temperature and refrigeration conditions and verified the reliability and accuracy of the sensor by utilizing changes in total volatile organic compound content. Then, two other machine learning algorithms, namely multi-layer perceptron (MLP) and support vector machine (SVM), are compared with the CNN model to compare their applicability and efficiency on multiple types of data. This approach can be extended to IoT applications [110]. Ferreira et al. used near infrared spectroscopy and electronic nose equipment for rapid, nondestructive testing of dragon fruit quality. Firstly, the near infrared spectrum or electronic nose data were used as predictors to classify the samples at different mature stages with high accuracy. Using near-infrared spectral data and partial least squares regression (PLSR) as predictive factors, the total titratable acidity and pH value of pitaya fruit were predicted, with *R*^2^ of 0.89 and 0.83 and RMSEP of 0.03 and 0.23, respectively. The electronic nose data and PLSR model were also used for TA and pH prediction, with *R*^2^ of 0.85 and 0.86 and RMSEP of 0.04 and 0.22, respectively. This work proposed a rapid non-destructive testing and classification technology for fruit and vegetable quality, which is different from the high cost and environmental protection of traditional analysis technology (GC-MS). The system has low cost, high working efficiency, considerable reliability, and a high environmental protection degree. The author also pointed out that the system can be used to predict TSS, moisture, and phenolic content and classify pitaya fruits based on their shelf life stages, which also provides new strategies and ideas for the preservation of other fruits and vegetables [111]. In another study, Ktenioudaki et al. used hyperspectral imaging technology combined with a partial least squares regression (PLSR) model to accurately predict the shelf life of strawberries. The author stored the harvested strawberries at five different temperatures for 9 days and graded them based on their appearance attributes (color, texture). Firstly, the author used the PLSR model to predict the appearance score of strawberries. The results showed that the PLSR model had high prediction accuracy, with an *R*^2^ of 0.97 and a root mean square error (RMSE) of 0.17. Afterwards, based on the appearance score of the PLSR model, the author developed a model using the Arrhenius equation to predict the remaining shelf life of strawberries, with an *R*^2^ of 0.86. In addition, the author also developed predictive models for other quality attributes and biochemical characteristics of strawberries, such as weight, ascorbic acid, and soluble solid content. The non-destructive system developed by the research institute can provide objective product quality assessment during the supply chain circulation process, help consumers and operators make decisions and take corresponding measures based on the product status, and minimize economic losses and resource waste as much as possible [112].

## 5. Outlook and Challenges

Food quality and safety have always been the focus of attention for consumers and business operators. In recent years, machine learning has shown strong potential in the fields of food quality control and food shelf life prediction. In the future, the research focus and development trend of machine learning in the fields of food preservation and food shelf life prediction may focus on the following aspects:

(1) Optimization and selection of model parameters

When building the fresh-keeping quality evaluation and shelf-life prediction model based on machine learning, the parameters of the model can be selected through the optimization algorithm. Determine the best conditions based on optimization to maximize shelf life in different processes. For example, the parameter penalty factor and kernel function width in support vector regression models can be optimized through particle swarm optimization and grey wolf optimization algorithms in group optimization algorithms and then brought into the model for training, greatly improving the accuracy of the model.

(2) Expansion of data samples and selection of models

Due to the short shelf life of certain foods, the number of physical and chemical indicators that can be measured for such foods will result in a small sample of model training data, which is one of the limitations of machine learning models. This requires researchers to find ways to expand the model training data samples to improve model accuracy. At the same time, we can compare the performance of multiple models in order to select appropriate machine learning models when dealing with small sample data in the future. Compared with neural network models such as CNN, traditional machine learning models such as SVM are more suitable for predicting small sample data.

(3) Multi-model fusion

In the future, the key influencing factors affecting food quality should be measured, and the multi-model fusion method should be used to achieve accurate assessment and prediction of food quality and shelf life, further improve the performance of the model, and reduce the drawbacks of the single model. By collaborating multiple machine learning models and combining their respective strengths, the overall accuracy of predictions can be improved. Joint learning can integrate data from different sources, utilize the expertise of each model in specific areas, and form more comprehensive prediction results. In addition, federated learning can improve the generalization ability of the model, enabling it to maintain high prediction accuracy in different contexts.

(4) The combination of deep learning and non-destructive testing technology

Deep learning, as a new branch of machine learning, can extract features from big data through networks, transform data using miscellaneous functions that can abstract and represent data at the hierarchical level, and achieve representation of input data. Non-destructive testing technologies such as electronic noses, UV-visible near-infrared spectroscopy, ultrasonic sensing, and visual sensing can monitor and gain insight into the quality status of food without damaging it. The integration of multiple non-destructive sensing technologies has enabled researchers to have a more accurate control and understanding of product status. Food information can also be used as input parameters of deep learning models, and the control accuracy of food information will affect the prediction accuracy of machine learning models to a certain extent. Therefore, the further integration of rapidly developing non-destructive sensing technology and deep learning is an effective strategy to improve model accuracy, ensure food quality, extend food shelf life, and save resources at present.

(5) Combination of deep learning and intelligent food supply chain system

In the future, we should make better use of the existing deep learning model to monitor and regulate the quality status and remaining shelf life of various products and integrate various parameters into the software system for online quality prediction [113]. Afterwards, it is combined with a blockchain-based food intelligent supply chain system to evaluate the status information of goods in real time and present it to operators and consumers so that operators can make the optimal marketing strategy for food status and consumers can make the most appropriate purchase decision. This not only solves the problem of low accuracy in predicting food freshness in the cold chain system, but also accelerates the construction of an intelligent agricultural system and effectively reduces resource waste.

(6) Machine learning combined with quantum computing

Machine learning can also be combined with quantum computing, utilizing the parallel computing power of quantum bits to accelerate the training process of machine learning models and reduce computation time. Meanwhile, the powerful processing capability of quantum computing can handle more complex datasets and improve the accuracy of predictions.

(7) The development of explainable AI (XAI) in the field of machine learning for predicting food quality

XAI has many advantages in solving the problem of machine learning black boxes. Firstly, by improving model transparency, such as through XAI technology and the development of the interactive tool ATMSeer, users can view and control the working system of automatic machine learning, making the decision-making process of machine learning models more transparent and promoting the foundation and logic behind non-expert food quality prediction. Secondly, it can enhance users’ trust; by providing detailed explanations of the predicted results through XAI, it can increase users’ trust in machine learning models that predict food quality. Finally, XAI can help optimize the decision-making process by helping non-experts better understand food quality prediction results and make wiser and more accurate decisions.

## 6. Conclusions

Machine learning models can efficiently and accurately detect defects and contaminants on the surface of food through technologies such as image recognition and computer vision, improve quality inspection efficiency and accuracy, and ensure that food meets safety standards; real-time monitoring of various parameters during food production and transportation; predicting and identifying potential quality issues; providing a scientific basis for optimizing production lines; and reducing the production of non-conforming products. Moreover, researchers can use machine learning algorithms to analyze key factors affecting shelf life, establish predictive models, accurately predict the shelf life of food, and provide an effective basis for the formulation of storage, circulation, and distribution strategies for products.

This article reviews the latest research on the application of machine learning in food quality control and shelf life prediction. Firstly, the definition and algorithm types of machine learning are elaborated, followed by a review of the application research of various algorithms in the field of food quality and shelf life prediction. Researchers use different models and algorithms for different types of fruits and vegetables. By taking different factors that affect food quality and shelf life during food storage as input parameters, the model is effectively trained in the process of testing and learning so that the model can achieve the final ideal accuracy. At present, researchers will also combine machine learning with various non-destructive testing technologies to regulate food quality and predict shelf life. This is particularly important for effectively ensuring food quality and extending food shelf life without damaging the food. Compared with other traditional dynamic models based on traditional food quality losses or microbial changes, the machine learning model has higher predictive accuracy because it has a large amount of data for training, validation, and prediction. Secondly, due to the strong self-learning ability of deep learning models, researchers can consider the factors affecting the prediction indicators more comprehensively and do not need to consider the specific chemical changes or internal causes in the process of food quality change. However, at the same time, this means that researchers cannot obtain any specific relationship between input indicators and predictive indicators from the machine learning model itself, so the interpretability of the machine learning model needs to be improved. Focusing on the interpretability of machine learning models not only helps to improve their credibility but also serves as a responsibility for model developers and users. By improving the interpretability of the model, it can enhance users’ confidence in the model and facilitate tracking and correction when problems arise, thereby solving the black box problem. In addition, the parameter adjustment and training degree during model training will affect the prediction accuracy of the model.

## Figures and Tables

**Table 1:** The advantages and disadvantages of traditional dynamic models and machine learning models.

Principle	Method	Predictor	Advantages	Disadvantages	Reference
Traditional Dynamics	Chemical kinetics	Total plate count (TPC)	Simple form, suitable for various shelf life predictions	Usually used in conjunction with the Arrhenius equation, only considering the effect of temperature on quality changes	[10]
	Microbial kinetics	Specific spoilage organism	In addition to temperature, the impact of environmental factors such as humidity and pH value on shelf life was also considered	Microbial indicators are highly correlated with changes in food quality and are not suitable for predicting the shelf life of fresh food with shorter storage times	[11]
Machine Learning	BP neural network, Support Vector Regression Machine	Sensory, physicochemical, and microbiological indicators	No need to understand the specific underlying principles that cause food quality decay, reducing errors caused by insufficient research on the principles	Unable to express and analyze the internal relationship between input and output	[12,13]

**Table 2:** Application progress of machine learning algorithms in food shelf life prediction.

Food Products	Machine Learning Algorithms	Predication Basis	Reference
Cauliflower	RF, XGBoost	Different packaging conditions and temperatures	[74]
fresh-cut green peppers	BP	O_2_, CO_2_, temperature, humidity	[75]
Table grape	BP, RBF	storage temperature, relative humidity, sensory average score, peel hardness, SSC, weight loss rate, rotting rate, fragmentation rate, and color difference	[76]
cherry tomatoes	ELM, PLSR	The data on e-nose	[77]
Strawberries	BP	O_2_, CO_2_, temperature, humidity	[78]
Fresh Date Fruits	RNN	pH, TSS, sugar, tannin, and MC	[79]
Citrus	ELM, RF, SVM	The data on Electronic Tongue and Electronic Nose	[80]
Banana	BP, RBF	image features and average spectra	[81]
Pears	CNN	hyperspectral imaging	[82]
quail eggs	PLS, SVM	NIR spectroscopy	[83]
blueberries	SVM, CNN	Ten physical and chemical flavor indices of blueberries (such as catalase, flavonoids, and soluble solids)	[63]
Persimmon	CNN, BP	RGB image	[84]
cherry tomatoes	PLS, SVM, ELM	NIR spectroscopy	[85]
sauerkraut	CNN	The photos of Sample from different periods	[86]
mushroom	SVM, ANN, PLS	Different packaging conditions and temperatures	[87]
Banana	ANN	Mobile image and appearance color characteristics	[88]
Large cranberry	ANN, SVM	storage time and storage temperatures	[89]
Strawberries	CNN	RGB image	[90]
Spinach	ANN, SVM	Digital images	[91]
Table grape	PLS, ANN	Near-infrared (NIR) spectroscopy	[92]

**Table 3:** Application of machine learning models in food quality control or food shelf life extension.

Food Products	Purpose of the Study	Input Indicator	Machine Learning Models	Reference
Spinach	The freshness identification of spinach preserved at different temperatures for different durations	Hyperspectral images	PLS, SVM, ELM	[100]
Strawberries	Evaluation of storage time	Visible/near-infrared hyperspectral images	PLS, RF, SVM	[101]
grape tomatoes	Estimate total soluble solids (TSS), titratable acidity (TA), and pH of the grape tomatoes	Fiber optic spectroscopy	PLS	[102]
banana slices	Predict moisture content, hardness, and fracturability of banana slices	Near-infrared hyperspectral images, chemometrics	PLS, SVM	[103]
carrot slices	Prediction of total carotenoids, color, and moisture content of carrot slices	Vis–NIR hyperspectral images	PLS	[104]
broccoli heads	Nondestructive prediction of the shelf life of broccoli heads	Hyperspectral images	ANN	[97]

## Data Availability

No new data were created or analyzed in this study. Data sharing is not applicable to this article.
